# Adolescent Sexual Violence in the Digital Era: A Case-Based Medico-Legal Study

**DOI:** 10.7759/cureus.100613

**Published:** 2026-01-02

**Authors:** Meryem Bouchalta, Hicham Benhadda, Bahia Belkaid, Ahmed Belhousse

**Affiliations:** 1 Forensic Medicine, Faculty of Medicine and Pharmacy, Sidi Mohamed Ben Abdellah University, Fez, MAR; 2 Forensic Medicine, Faculty of Medicine and Pharmacy, Ibn Zohr University, Agadir, MAR; 3 Forensic Medicine, Faculty of Medicine and Pharmacy, Centre Hospitalier Universitaire Mohammed VI, Oujda, MAR; 4 Forensic Medicine, Faculty of Medicine and Pharmacy, Hassan II University of Casablanca, Casablanca, MAR

**Keywords:** adolescents, cyber-violence, digital context, forensic medicine, psychological impact, sexual violence

## Abstract

The rapid expansion of digital technologies has profoundly altered the contexts in which adolescents are exposed to certain forms of violence. Based on a six-month retrospective medico-legal study, this work examines cases of sexual violence involving minors, focusing on the role played by digital practices in the occurrence and progression of the events. The analysis reveals a range of configurations in which digital tools intervene at different stages of victimization trajectories. Psychological and social impact was frequently substantial, often contrasting with limited or absent physical findings at clinical examination. These findings highlight the relevance of a comprehensive medico-legal approach that systematically integrates the digital dimension in order to improve the identification and understanding of these complex situations among adolescents.

## Introduction

Sexual violence against minors represents a major public health and child protection concern, with long-lasting medical, psychological, and social consequences. Adolescence, a developmental period marked by profound identity and relational changes, constitutes a particularly high-risk phase of exposure to such violence, which may occur in complex and multifactorial contexts [1].

Concurrently, the rapid expansion of digital technologies and social media has profoundly transformed adolescents' modes of interaction, giving rise to new forms of victimization commonly referred to as cyber-violence. This term encompasses online harassment, sextortion, non-consensual dissemination of intimate images, and remote sexual exploitation, whose impact extends beyond the virtual sphere [2].

Several studies have shown that these digitally mediated forms of violence may be associated with significant psychological consequences, sometimes reported as comparable to those observed in offline violence [3].

While the international literature has extensively examined cyber-violence from psychosocial and educational perspectives, data from the medico-legal field remain limited, particularly regarding the interactions between cyber-violence and sexual violence. Medico-legal assessment faces specific challenges, including the frequent absence of objective physical findings and the underreporting of incidents, especially when they involve intimate digital content perceived as private by victims [4,5].

In this context, the analysis of medico-legal case files represents a valuable approach to explore how cyber-violence and sexual violence may intersect during adolescence and to document their clinical and psychosocial implications.

The present study adopts this perspective by providing a descriptive and analytical examination of sexual violence cases involving adolescents, with a particular focus on the role of cyber-violence within these victimization trajectories.

## Materials and methods

Study design and setting

This retrospective, descriptive, and analytical study was conducted in the Forensic Medicine unit of Hassan II University Hospital in Fez over a six-month period, from January to June 2025. The analysis was based on a retrospective review of medico-legal files compiled as part of routine clinical practice and outside the framework of any prospective research protocol.

Study population

During the study period, 180 minors referred for sexual violence were examined. Among them, 97 adolescents constituted the study population, defined according to the World Health Organization criteria as individuals aged 10-19 years. Within this adolescent subgroup, 77 individuals reported exposure to at least one form of cyber-violence, which occurred in the context of sexual violence.

Inclusion and exclusion criteria

Included were medico-legal files of adolescents who were victims of sexual violence and presented documented elements of cyber-violence, with sufficient clinical and forensic data for analysis. Files that were incomplete or did not allow the characterization of an interaction with digital technologies were excluded.

Data collection

Data were extracted from medico-legal certificates, clinical examination reports, and medico-legal interviews conducted with the victims. When available, information provided by parents or competent authorities was also considered. The variables analyzed included sociodemographic characteristics, the nature of the reported events, the type of cyber-violence involved, the interaction between cyber-violence and sexual violence, clinical findings, and psychological, educational, and social impact.

Definition of cyber-violence

Cyber-violence was defined as any intentional harmful act carried out through a digital tool or electronic communication network, including cyberbullying, blackmail and sextortion, non-consensual dissemination of intimate content, and forms of remote sexual exploitation, in accordance with the definition provided in the Introduction.

Data analysis

Data analysis was primarily descriptive and was complemented by a qualitative review of the medico-legal files. This approach enabled the identification of three main patterns of interaction between cyber-violence and sexual violence. Selected clinical observations were included for their illustrative value with respect to these patterns, without aiming for narrative exhaustiveness.

Ethical considerations

This study was conducted in accordance with the ethical principles applicable to retrospective research in forensic medicine and consisted of a retrospective descriptive analysis of anonymized medico-legal records. According to institutional policy, formal institutional review board approval was not required for this type of study. Informed consent for the medico-legal examination and for the use of anonymized data for scientific purposes was obtained from the minors and their legal representatives. All data were strictly anonymized and handled in compliance with ethical standards to ensure confidentiality, non-identifiability of the victims, and data protection.

## Results

Study population

During the study period, 180 minors who were victims of sexual violence were examined at the forensic medicine unit. Among them, 97 were adolescents aged 10-19 years, who constituted the study population. Within this adolescent subgroup, exposure to at least one form of cyber-violence was identified in 77 adolescents (79.4%), while 20 adolescents (20.6%) had no documented exposure to cyber-violence.

Females accounted for 54 adolescents (70.1%) exposed to cyber-violence, while males represented 23 adolescents (29.9%). All adolescents were enrolled in school prior to the events (77 cases, 100%), with 50 (64.9%) attending middle school and 27 (35.1%) high school. Demographic and clinical characteristics are summarized in Table 1.

**Table 1:** Demographic and clinical characteristics of adolescents exposed to cyber-violence (n=77) *Other forms include cyberharassment, digital blackmail, and non-consensual dissemination of intimate content.

Characteristic	n (%)
Sex	
Female	54 (70.1)
Male	23 (29.9)
Age group	
Adolescents (10-19 years)	77 (100)
Educational status before events	
Enrolled in school	77 (100)
Middle school	50 (64.9)
High school	27 (35.1)
Type of violence	
Sexual violence with physical contact	59 (76.6)
Isolated cyber-violence (no physical contact)	14 (18.2)
Physical findings at examination	
Objective lesions present	35 (45.5)
No objective lesions	42 (54.5)
Pattern of interaction between cyber-violence and sexual violence	
Cyber-violence as a consequence of sexual violence	29 (37.7)
Cyber-violence as a triggering/facilitating factor	12 (15.6)
Cyber-violence as a means of prolongation/aggravation	36 (46.7)
Type of cyber-violence	
Sextortion	37 (48.1)
Other forms*	40 (51.9)
Psychological and social impact	
Psychological impact reported	77 (100)
Suicide attempts	5 (6.5)
Runaway episodes	7 (9.1)
Educational consequences	
Temporary school interruption with resumption	15 (19.5)
Definitive school dropout	6 (7.8)
Digital forensic investigation performed	
Yes	17 (22.1)
No	60 (77.9)

Patterns of interaction between cyber-violence and sexual violence

Analysis of the medico-legal files of the 77 adolescents exposed to cyber-violence allowed the identification of three main patterns of interaction between cyber-violence and sexual violence.

Cyber-violence occurring as a consequence of sexual violence was observed in 29 adolescents (37.7%). Cyber-violence acting as a triggering or facilitating factor was identified in 12 adolescents (15.6%). Cyber-violence used as a means of prolongation or aggravation of sexual violence was the most frequent pattern, observed in 36 adolescents (46.7%).

Isolated cyber-violence without associated physical sexual assault was identified in 14 adolescents (18.2%).

Types of cyber-violence

Sextortion was the most frequently identified form of cyber-violence, accounting for 37 adolescents (48.1%). Other forms, including cyberharassment, digital blackmail, and non-consensual dissemination of intimate content, were identified in 40 adolescents (51.9%), with relatively comparable frequencies across these categories.

Medico-legal findings

Sexual violence involving physical contact was reported in 59 adolescents (76.6%). However, objective physical lesions were identified during clinical examination in only 35 adolescents (45.5%). No physical injuries were observed in cases involving isolated cyber-violence.

Psychological, social, and educational impact

A psychological impact was reported in all adolescents exposed to cyber-violence (77 cases, 100%), predominantly characterized by anxiety and depressive symptoms associated with impaired social functioning.

Suicide attempts were documented in five adolescents (6.5%), including one male and four females. Suicidal ideation without a documented attempt was reported in eight adolescents (10.4%). Runaway episodes were observed in seven adolescents (9.1%), exclusively among females, with a predominance of cases occurring in rural settings.

Regarding educational outcomes, temporary interruption of schooling with subsequent resumption was observed in 15 adolescents (19.5%), while definitive school dropout was documented in six adolescents (7.8%).

Digital forensic elements

Digital forensic analysis of electronic devices conducted by police services in Fez was performed in 17 cases (22.1%), allowing the identification of probative digital elements supporting the reported facts, particularly when physical findings were absent or limited.

## Discussion

Cyber-violence represents an emerging phenomenon within adolescents' digital environments, whose implications for sexual violence remain insufficiently characterized, particularly in the medico-legal field. The widespread use of social media and electronic communication tools exposes this population to specific forms of vulnerability, the consequences of which extend beyond the strictly technological sphere and become embedded in complex trajectories of coercion, victimization, and violence.

In our series, a high proportion of adolescents who were victims of sexual violence reported a direct involvement of digital tools either in the occurrence of the events or in their subsequent evolution. This finding suggests a close interrelationship between the digital sphere and sexual violence. In this context, medico-legal data remain limited, highlighting the contribution of the present study to the understanding of these situations among adolescents.

Cyber-violence, psychological vulnerability, and challenges in detection

Adolescence represents a critical period of identity formation, characterized by heightened sensitivity to peer perception, stigmatization, and social exclusion. In this context, cyber-violence, owing to its public nature, persistence over time, and limited controllability, amplifies mechanisms of shame, guilt, and fear, contributing to sometimes severe psychological impact, independent of the clinical severity of the acts. Several studies have shown that adolescents exposed to cyber-violence present high rates of anxiety disorders, depressive symptoms, and suicidal behaviors, comparable to or even exceeding those observed in offline forms of violence [6,7]. This vulnerability is particularly pronounced when cyber-violence involves a sexual dimension, notably in situations of sextortion, non-consensual dissemination of intimate images, or pedopornographic exploitation, which are associated with the most severe psychological consequences [8].

International epidemiological data report prevalences of cyber-violence among adolescents ranging from 10% to 40%, depending on the definitions and methodologies used [9]. However, these figures are likely to underestimate the true extent of the phenomenon, particularly for sexually related forms, which are frequently associated with a low propensity for disclosure. The literature on sexual violence against minors indicates that disclosure is often delayed, partial, or absent, due to shame, fear of judgment, guilt, or the perception of the medical professional as not concerned with such issues [10]. This reluctance may be explained by the fact that many minors perceive medico-legal consultations as primarily focused on physical examination and the issuance of a certificate and therefore do not spontaneously identify digital violence as falling within the medical or medico-legal domain. Underreporting is further reinforced when the content was initially shared consensually within an emotional relationship, a situation frequently associated with confusion between responsibility and victimization, leading to reluctance to seek medical or judicial assistance [11].

Moreover, several authors emphasize that healthcare and medico-legal professionals, although aware of the concept of cyber-violence, do not systematically explore the digital dimension during the assessment of minors who are victims of sexual violence, thereby contributing to the under-identification of these situations [12]. In our experience, the implementation of systematic questioning regarding cyber-violence revealed a substantial number of situations that had not been spontaneously disclosed by victims. In addition, some adolescents did not perceive cyber-violence as falling within the medical or medico-legal field, while others failed to mention it due to trivialization or simple omission in the context of everyday digital use.

Moroccan and international legal framework for digital sexual violence against minors

International legal instruments and policy frameworks recognize the need to protect children and adolescents from sexual exploitation and abuse, including acts facilitated by information and communication technologies. In this context, national legal frameworks play a central role in the effective regulation and medico-legal management of such offenses.

In Morocco, sexual violence against minors is primarily regulated by the provisions of the Moroccan Penal Code, notably Articles 484-490, which criminalize sexual offenses against minors as well as offenses related to child pornography, including the production, possession, and dissemination of sexually explicit content involving minors [13]. The legal recognition of offenses committed through electronic means has been strengthened by Law No. 03-07 on electronic exchange of legal data and Law No. 09-08 on the protection of individuals with regard to the processing of personal data, which strictly regulates the collection, storage, and dissemination of personal data, including intimate images and digital content, in the absence of valid consent when a minor is involved [14].

Within this framework, the non-consensual dissemination of intimate images, including when associated with blackmail or sextortion, constitutes a criminal offense linked to violations of privacy, dignity, and moral integrity. However, as highlighted in the literature, legal frameworks often lag behind the rapid evolution of digital practices, making the legal qualification and objective assessment of such acts complex, particularly in the absence of objective physical injuries [15].

In this context, the forensic examination of digital devices plays a central role. Conducted by specialized units of the General Directorate of National Security (DGSN) in accordance with the provisions of the Moroccan Code of Criminal Procedure, such examinations enable the extraction, analysis, and preservation of digital evidence (images, videos, messages, metadata), which frequently constitute decisive probative elements in judicial proceedings related to digital sexual violence against minors [16-18].

Role of the forensic physician

The forensic physician occupies a central position at the interface of healthcare, the judicial system, and prevention. Their role is not limited to the identification of physical injuries, which may be absent, but extends to the identification of situations involving cyber-violence through targeted and appropriate questioning, the assessment of psychological and social impact, the integration of reported digital elements into the medico-legal narrative and conclusions, with the aim of guiding judicial authorities toward appropriate technical forensic investigations, and the early referral of victims to suitable medico-psychological care.

Forensic medicine units, therefore, constitute privileged observatories of these emerging forms of violence, as they provide access to objective clinical and contextual data that are essential for understanding these phenomena and for informing prevention and protection strategies.

Limitations of the study and future perspectives

This study highlights the diversity of interactions between cyber-violence and sexual assaults among minors, illustrated by representative clinical observations drawn from forensic practice. Its limitations lie in its retrospective nature, the absence of standardized psychological assessment tools, as well as the sample size and the relatively short duration of the study period, which may restrict the generalization of the results.

However, these limitations are consistent with the exploratory nature of this work, which represents a first descriptive approach to an emerging phenomenon within the Moroccan forensic context. Moreover, this study is part of a broader research initiative, with a larger-scale investigation currently underway to further deepen the epidemiological and clinical analysis of the interactions between cyber-violence and sexual violence among minors.

## Conclusions

Sexual violence against adolescents in the digital era presents complex medico-legal challenges that extend beyond traditional clinical frameworks. This study highlights the central role of digital practices in shaping contemporary trajectories of victimization, often amplifying psychological and social harm despite limited or absent physical findings. Integrating the digital dimension into medico-legal assessment is therefore essential to improve the identification, understanding, and prevention of these forms of violence. Forensic medicine units occupy a strategic position in addressing this emerging phenomenon and in contributing to more effective medico-judicial responses for the protection of minors.
